# Epidemiology and Short-Term Outcomes of Acute Coronary Syndrome in a Nigerian Tertiary Hospital: A Prospective Cohort Study

**DOI:** 10.7759/cureus.104005

**Published:** 2026-02-20

**Authors:** Akinwumi Ojo, Osagioduwa Mike Atoe-Imagbe, Ifunanya Agu-Jefferson, Ehi Ogbemudia, Aisosa Ogbomo, Veronica Josephs, Austine Obasohan

**Affiliations:** 1 Department of Internal Medicine/Cardiology, University of Benin Teaching Hospital, Benin City, NGA; 2 Department of Medicine/Cardiology, Betsi Cadwaladr University Health Board, Bangor, GBR; 3 Department of Medicine, Delta State University Teaching Hospital, Oghara, NGA; 4 Department of Acute/General Medicine, North Cumbria Integrated Care, West Cumberland Hospital, Whitehaven, GBR; 5 Department of Medicine/Cardiology, University of Benin Teaching Hospital, University of Benin, Benin City, NGA; 6 Department of Internal Medicine, University of Benin Teaching Hospital, Benin City, NGA; 7 Department of Medicine, College of Medical Sciences, University of Benin, Benin City, NGA

**Keywords:** acute coronary syndrome, mortality, nigeria, short-term outcomes, stemi, troponin

## Abstract

Background: Acute coronary syndrome (ACS) is associated with significant morbidity and mortality, with outcomes influenced by disease subtype and patient comorbidities. Data on the epidemiology and outcomes of ACS in Nigeria remain limited.

Objectives: This study aimed to describe the epidemiological characteristics, clinical presentation, and short-term outcomes of patients with ACS in a Nigerian tertiary hospital.

Methods: This was a prospective cohort study conducted at the University of Benin Teaching Hospital (UBTH), Benin City, Nigeria, from May 11, 2022, to May 10, 2023. Thirty adult patients (n = 30) aged ≥18 years with a confirmed diagnosis of ACS were consecutively recruited and followed for three months. Data on sociodemographic characteristics, cardiovascular risk factors, clinical features, electrocardiographic and echocardiographic findings, and treatment outcomes were collected. Statistical analysis was performed using SPSS version 27 (IBM Corp., Armonk, NY), with statistical significance set at p < 0.05.

Results: The mean age of the patients was 59.0 ± 14.2 years, with the majority aged ≥60 years (19 patients, 63.3%). There were 20 male patients (66.7%) and 10 female patients (33.3%). The most prevalent cardiovascular risk factor was hypertension (22 patients, 73.3%), followed by alcohol intake (12 patients, 40%), dyslipidemia (six patients, 20%), and diabetes mellitus (five patients, 16.7%).

ST-segment elevation myocardial infarction (STEMI) was the most common ACS subtype (16 patients, 53.3%), followed by unstable angina (nine patients, 30%) and non-ST-segment elevation myocardial infarction (NSTEMI) (five patients, 16.7%).

Overall mortality was seven patients (23.3%), comprising in-hospital deaths in five patients (16.7%) and out-of-hospital deaths within three months in two patients (6.7%). Mortality was higher among patients aged ≥60 years (six of seven deaths, 85.7%) and among those with STEMI (six of seven deaths, 85.7%). Elevated cardiac troponin T levels were significantly associated with increased mortality (seven of seven deaths, 100%, p = 0.010).

Conclusion: Acute coronary syndrome predominantly affected older male patients, with hypertension and alcohol use being the most common risk factors. STEMI was the most frequent presentation and accounted for most deaths. The high short-term mortality rate in the index study underscores the need for improved early diagnosis, risk factor modification, and access to timely reperfusion therapies to improve outcomes.

## Introduction

Acute coronary syndrome (ACS) represents a major cause of morbidity and mortality worldwide and encompasses a spectrum of clinical conditions, including unstable angina, non-ST-segment elevation myocardial infarction (NSTEMI), and ST-segment elevation myocardial infarction (STEMI) [1,2]. Death from ACS has been found to occur at comparatively younger ages in low- and middle-income countries (LMICs) than in high-income countries (HICs). Although HICs continue to record significant mortality from ACS, more than half of all ACS-related deaths occur in LMICs [3].

Economic growth and lifestyle changes have been attributed to the increasing prevalence of ischemic heart disease (IHD) risk factors. Despite the global reduction in smoking prevalence since 1980, the absolute number of smokers has risen, particularly in LMICs [4]. Likewise, the consumption of unhealthy foods such as sugary beverages, processed foods, and alcohol has increased, and physical inactivity levels in many LMICs are now comparable to those in HICs [5].

Recent studies report that approximately 1.2 million hospital discharges in the United States are associated with an ACS diagnosis annually, and about 20% of patients are rehospitalized for ischemic heart disease within a year [6]. The management of ACS carries substantial financial implications, with an estimated annual cost of about $150 billion in the United States, equivalent to approximately ₦225-₦255 trillion, encompassing both direct and indirect expenses. Invasive procedures such as coronary angiography and revascularization account for a significant proportion of healthcare costs [7].

While the burden of ACS is well-documented in Western populations, evidence from sub-Saharan Africa remains limited. The epidemiology, risk profile, and outcomes in this region may differ due to limited resources and variable access to advanced cardiac care [8,9]. The increasing incidence of ACS in sub-Saharan Africa has been linked to the adoption of Western lifestyles, urbanization, and population aging, factors contributing to a higher prevalence of cardiovascular risk factors [10].

Empirical evidence suggests that despite the rising incidence of ACS in sub-Saharan Africa, management remains suboptimal. Limited access to advanced cardiac care contributes to delays in diagnosis and treatment, leading to increased morbidity and mortality. For instance, a study from Ethiopia reported that 72.6% of cardiovascular-related deaths were due to STEMI, with an in-hospital mortality rate of 27.4% [11].

In LMICs, the management of ACS faces challenges across all levels of healthcare delivery. These include delayed presentation, poor adherence to evidence-based timelines for reperfusion therapy, and limited availability of revascularization facilities. Other factors, such as lack of healthcare financing, suboptimal medication adherence, and inadequate lifestyle modification, further hinder optimal outcomes [12].

In Nigeria, ischemic heart disease (IHD) was once considered rare but is now recognized as an increasing cause of hospitalization and mortality [13,14]. The rising burden of modifiable cardiovascular risk factors, such as hypertension, diabetes mellitus, dyslipidemia, and alcohol consumption, further amplifies the public health significance of ACS [15-17]. Historically, the prevalence of IHD in Nigeria was very low. Falase et al. reported only 26 IHD cases over a 10-year period (1961-1970), corresponding to an incidence of one in 20,000 hospitalized adults [18]. A similar outcome was also observed by Josephs at the University of Benin Teaching Hospital (UBTH), who identified 26 acute myocardial infarction (AMI) cases over a 10-year period (1998-2007), with a frequency of 0.4 per 10,000 hospital admissions [19].

Despite these emerging trends, data on the clinical spectrum and short-term outcomes of ACS from Nigerian tertiary hospitals remain sparse. This study, therefore, aimed to describe the epidemiological and clinical characteristics of patients admitted with acute coronary syndrome (ACS) in a tertiary hospital in southern Nigeria and to evaluate short-term outcomes over a three-month follow-up period, specifically assessing in-hospital mortality, post-discharge mortality, readmission rates, and duration of hospitalization.

## Materials and methods

This was a prospective cohort study conducted at the University of Benin Teaching Hospital (UBTH), Benin City. UBTH is a Federal Government-owned tertiary hospital located in Benin City, the capital of Edo State in the South-South region of Nigeria. It is a 900-bed capacity facility that provides tertiary healthcare services and serves as a major referral center in the area. The study was conducted from May 11, 2022, to May 10, 2023.

Adult patients aged 18 years and above with a confirmed diagnosis of acute coronary syndrome (ACS) were consecutively recruited into the study. The exclusion criteria included patients presenting with angina symptoms who did not meet the diagnostic criteria for ACS, patients with a prior history of ACS admitted for unrelated conditions, and patients who developed ACS postoperatively following percutaneous coronary intervention (PCI) or coronary artery bypass graft (CABG) surgery. Patients received standard-of-care treatment according to contemporary guidelines for acute coronary syndromes. Thrombolytic therapy was administered to eligible patients presenting within the recommended time window and without contraindications. Percutaneous coronary intervention (PCI) was available at the tertiary hospital during the study period. All patients were prescribed guideline-directed medical therapy (GDMT) as indicated, including antiplatelet agents, statins, beta-blockers, and angiotensin-converting enzyme (ACE) inhibitors/angiotensin receptor blockers (ARBs). Details of therapy initiation, dosing, and adherence were documented from medical records.

The sample size was estimated using Fisher’s formula for single proportions. A prevalence rate of acute coronary syndrome in Nigeria of 1.6% was applied [20], with a 95% confidence level and a precision of 0.05. This yielded a minimum sample size of 24. After adjusting for a 10% attrition rate, a total of 30 participants were recruited. Eligible subjects who met the inclusion criteria were consecutively enrolled until the target sample size was achieved. The diagnosis of ACS was based on the 2014 American Heart Association (AHA)/American College of Cardiology (ACC) guidelines [21], incorporating clinical presentation, electrocardiographic (ECG) changes, and elevation of cardiac biomarkers (troponin T).

Data collected included sociodemographic characteristics, cardiovascular risk factors, clinical presentation, ECG patterns, echocardiographic findings, and treatment outcomes. Participants were followed for three months after discharge. Follow-up data were obtained through scheduled outpatient clinic visits. For patients who did not attend clinic appointments, structured telephone interviews were conducted using contact details provided at enrollment. In cases where participants were unreachable, hospital medical records and information from next of kin were reviewed to ascertain survival status and readmission events. Patients were followed up for short-term outcomes, including in-hospital mortality and duration of hospitalization. Echocardiography was performed during admission and repeated three months post-discharge to assess for new regional wall motion abnormalities and complications [22].

Operational definitions

Hypertension was defined as a prior physician diagnosis of hypertension, current use of antihypertensive medication, or documented blood pressure ≥ 140/90 mmHg on at least two occasions during admission [16].

Diabetes mellitus was defined as a prior diagnosis, current use of glucose-lowering medication, fasting blood glucose ≥ 126 mg/dL, or HbA1c ≥ 6.5%, in accordance with American Diabetes Association (ADA) criteria [17].

Dyslipidemia was defined as total cholesterol ≥ 200 mg/dL, low-density lipoprotein (LDL) cholesterol ≥ 130 mg/dL, high-density lipoprotein (HDL) cholesterol < 40 mg/dL (men) or HDL cholesterol < 50 mg/dL (women), triglycerides ≥ 150 mg/dL, or current use of lipid-lowering therapy [17].

Alcohol intake was defined as regular consumption of alcoholic beverages at least once weekly within the preceding six months [17].

Smoking was defined as current use of tobacco products or cessation within the previous 12 months [17].

Elevated troponin T and creatine kinase-MB (CK-MB) were defined as values above the 99th percentile upper reference limit of the assay used at the study center [17].

Chronic kidney disease (CKD) was defined as documented estimated glomerular filtration rate (eGFR) < 60 mL/min/1.73m² for ≥3 months or a prior physician diagnosis [17].

Data analysis was performed using IBM SPSS Statistics version 27 (IBM Corp., Armonk, NY). Missing data were minimal (<5%) and were handled using complete-case analysis. No imputation techniques were applied due to the small sample size. Continuous variables were presented as means ± standard deviations (SD) or as medians with interquartile ranges (IQR), depending on data distribution. Comparisons of normally distributed continuous variables between survivors and non-survivors (e.g., age, renal function, lipid profile, and fasting blood glucose) were conducted using Student’s independent t-test, while non-normally distributed continuous variables between the two groups were compared using the Mann-Whitney U test. Comparisons of non-normally distributed continuous variables across more than two independent groups (e.g., length of hospital stay among ACS subtypes) were performed using the Kruskal-Wallis H test. Categorical variables were summarized as frequencies and percentages. Associations between categorical variables and mortality, including age group, sex, cardiovascular risk factors, comorbidities, electrocardiographic findings, echocardiographic parameters, cardiac enzyme status, and ACS classification, were assessed using Fisher’s exact test due to small cell counts. Pearson’s chi-square test, with Yates’ continuity correction where appropriate, was used to evaluate associations between ACS subtypes and gender. Pearson’s correlation coefficient was calculated to assess the relationship between length of hospital stay and cardiac enzyme levels (troponin T and creatine kinase-MB). Odds ratios (OR) with 95% confidence intervals (CI) were computed to quantify the strength of associations between selected clinical variables and mortality. Due to the limited sample size and small number of outcome events (n = 7 deaths), multivariable logistic regression modeling was not performed to avoid model overfitting and unstable estimates. Instead, univariate analyses were conducted to explore associations between clinical variables and mortality. Findings should therefore be interpreted as exploratory. A p-value < 0.05 was considered statistically significant.

Ethical approval for the study was obtained from the UBTH Health Research Ethics Committee (approval number: ADM/E22/A/VOL.VII/148312102). Informed consent was obtained from all participants prior to recruitment. The study adhered to the ethical principles outlined in the Declaration of Helsinki (2013 revision) [23].

## Results

Sociodemographic characteristics

The mean age of the patients with ACS in this study was 59.0 ± 14.2 years, with an age range of 32-81 years, and the majority were aged 60 years and above. They included 20 (66.7%) male patients and 10 (33.3%) female patients with a male:female ratio of 2: 1 (Table 1).

**Table 1:** Sociodemographic characteristics of the study population SD: standard deviation

Variable	Male (n = 20)	Female (n = 10)	Total (N = 30)
Age (years)			
Mean ± SD	59.1 ± 14.7	59.1 ± 14.0	59.0 ± 14.2
Range	32-81	40-78	32-81
Age group (years), number (%)			
<60	8 (40)	3 (30)	11 (36.7)
≥60	12 (60)	7 (70)	19 (63.3)
Educational status, number (%)			
Non-formal	0 (0)	1 (10)	1 (3.3)
Primary	3 (15)	2 (20)	5 (16.7)
Secondary	3 (15)	2 (20)	5 (16.7)
Tertiary	14 (70)	5 (50)	19 (63.3)
Religion, number (%)			
Christian	20 (100)	10 (100)	30 (100)
Marital status, number (%)			
Single	1 (5)	0 (0)	1 (3.3)
Married	18 (90)	9 (90)	27 (90)
Widow/widower	1 (5)	1 (10)	2 (6.7)

CVD risk factors based on gender

The distribution of the CVD risk factors based on gender in the study population is shown in Table 2. Hypertension, dyslipidemia, alcohol intake, diabetes, heart diseases, and smoking were more prevalent in male patients, representing 56.7%, 13.3%, 16.7%, 10%, and 10% of the study population, respectively.

**Table 2:** CVD risk factors based on gender CVD: cardiovascular disease

CVD risk factors	Male (n = 20)	Female (n = 10)	Total (N = 30)	Statistical test	P-value
Hypertension	17 (85)	5 (50)	22 (73.3)	Fisher’s exact test	0.078
Dyslipidemia	4 (20)	2 (20)	6 (20)	Fisher’s exact test	1.000
Diabetes mellitus	5 (25)	0 (0)	5 (16.7)	Fisher’s exact test	0.140
Smoking	3 (15)	0 (0)	3 (10)	Fisher’s exact test	0.532
Alcohol intake	10 (50)	2 (20)	12 (40)	Fisher’s exact test	0.235
Heart diseases	3 (15)	0 (0)	3 (10)	Fisher’s exact test	0.532

Distribution of ACS based on gender

The distribution of the various classes of acute coronary syndrome based on gender is shown in Table 3. The majority (12, 60%) of the male patients had STEMI, while the most common class of ACS in the female patients is unstable angina. Five (50%) of the female patients had unstable angina, followed by STEMI in 40% of the population. The differences in the distribution were not statistically significant (p = 0.510).

**Table 3:** Distribution of ACS based on gender *Adjusted chi-square STEMI: ST-segment elevation myocardial infarction, NSTEMI: non-ST-segment elevation myocardial infarction, UA: unstable angina

	Male (n = 20)	Female (n = 10)	Total (N = 30)	Statistical test	P-value
STEMI	12 (60)	4 (40)	16 (53.3)	χ^2^ = 1.345*	0.510
NSTEMI	4 (20)	1 (10)	5 (16.7)	-	-
UA	4 (20)	5 (50)	9 (30)	-	-

Treatment outcome

**Figure 1:**
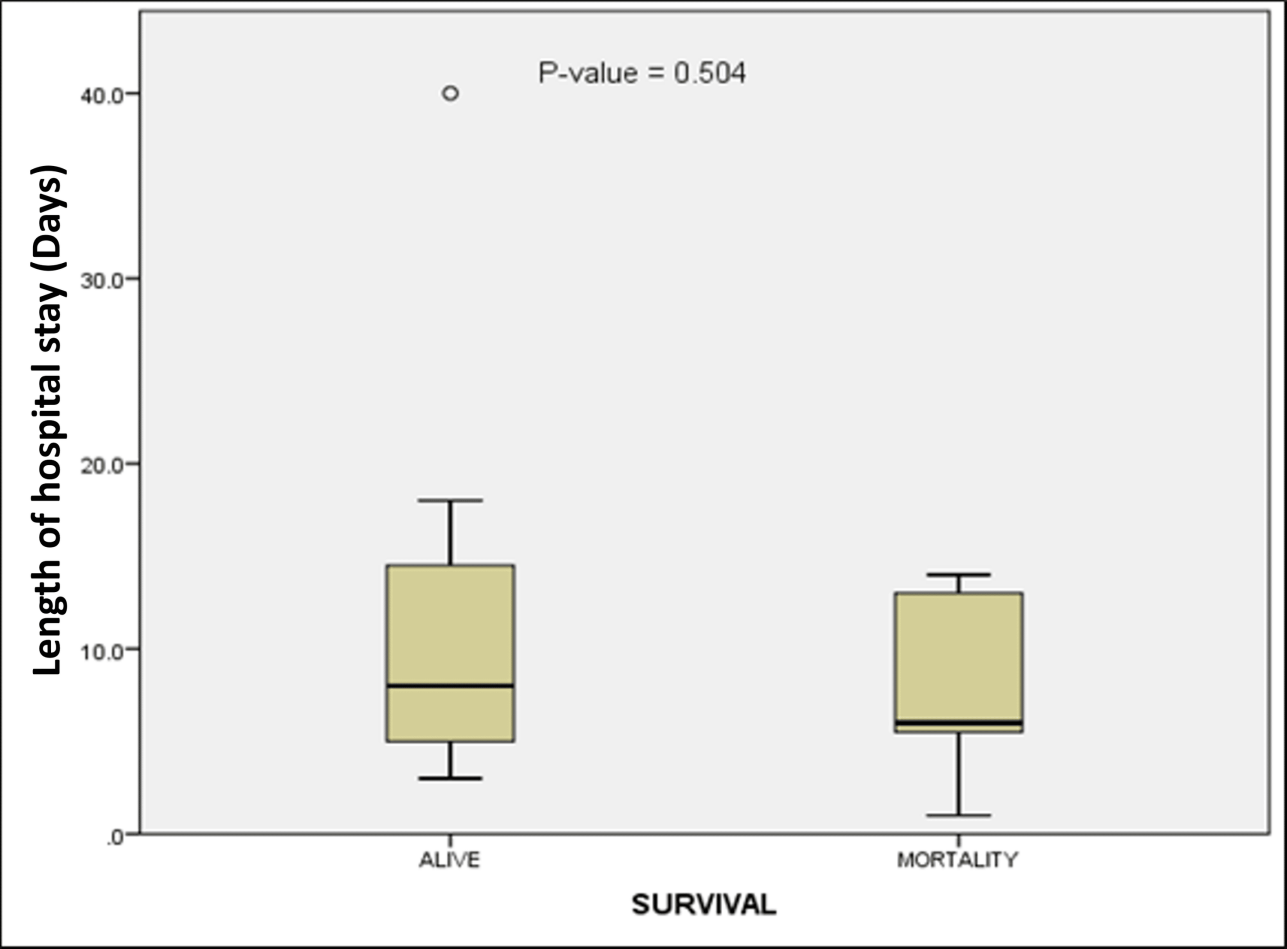
Length of hospital stay based on survival of the study population Length of hospital stay among survivors (n = 23, 76.7%) and non-survivors (n = 7, 23.3%) Statistical test: Mann-Whitney U test

There were seven mortalities during the study, accounting for 23.3% of the population. This included five (16.7%) in-hospital deaths and two (6.7%) out-of-hospital deaths. The median length of hospital stay was 7.5 days (interquartile range: 5-14 days). The duration of hospital stay was longer in survivors, but the difference in median was not statistically significant (6 (IQR: 5-15 days) versus 8 (IQR: 5-15); p = 0.504) (Figure 1).

**Figure 2:**
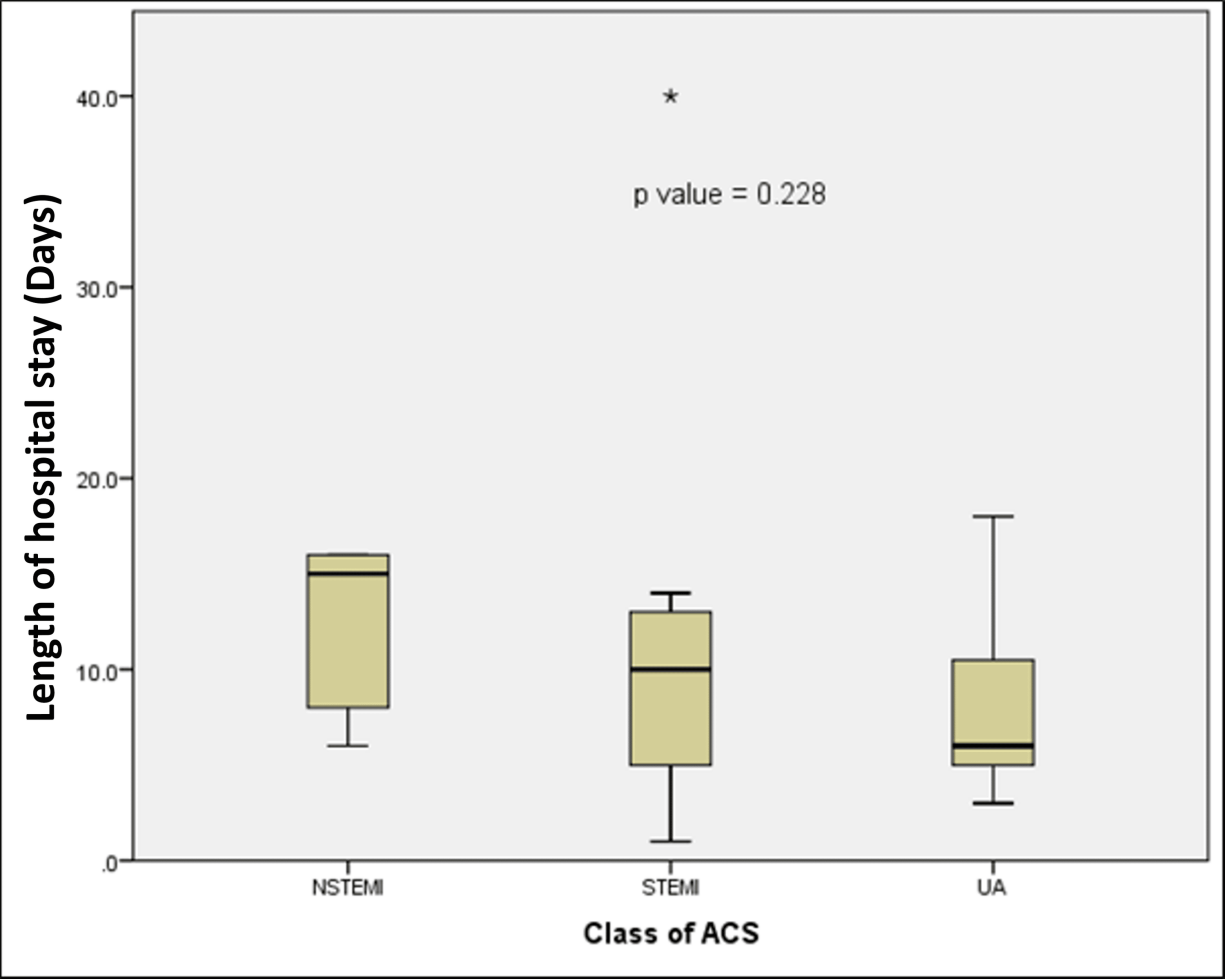
Length of hospital stay based on classification of ACS This figure illustrates mortality across the different acute coronary syndrome subtypes: STEMI (n = 16, 53.3%), NSTEMI (n = 5, 16.7%), and UA (n = 9, 30%). Statistical test: Kruskal-Wallis H test STEMI: ST-segment elevation myocardial infarction, NSTEMI: non-ST-segment elevation myocardial infarction, UA: unstable angina

The length of hospital stay was longest in the NSTEMI group (median: 15 (IQR: 7-16) days), followed by STEMI (median: 10 (IQR: 5-13.5) days), and the shortest was unstable angina (median: 6 (IQR: 5.0-12.3) days). The difference in median was not statistically significant (p = 0.228) (Figure 2).

Readmission

One (3.3%) of the study subjects was readmitted within the first 30 days of the event.

Correlation of length of hospital stay and cardiac enzyme levels

**Figure 3:**
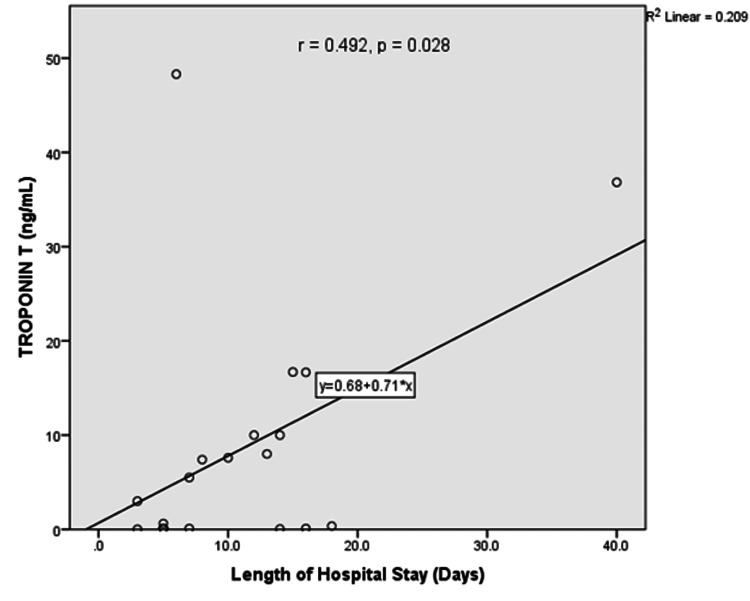
Linear graph showing correlation between troponin T and length of hospital stay The relationship between cardiac troponin T levels and treatment outcome was assessed using Pearson’s correlation coefficient.

**Figure 4:**
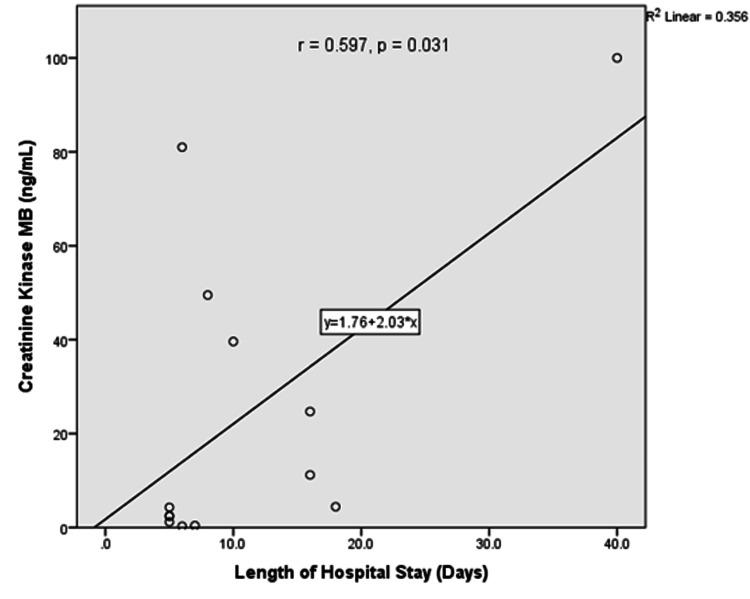
Correlation between CK-MB and length of hospital stay The relationship between CK-MB level and length of hospital stay was assessed using Pearson’s correlation coefficient. CK-MB: creatine kinase-MB

There were positive correlations between length of hospital stay and troponin T levels (r = 0.492, 0.028) and creatine kinase-MB (r = 0.597, p = 0.031). Figures 3 and Figure 4 show the linear graph for length of hospital stay and troponin T, and length of hospital stay and CK-MB, respectively.

Associations between age, sex, and mortality

Six (85.7%) of the mortality were in persons 60 years and above. Age 60 and above is associated with high odds of mortality, although not statistically significant (OR: 4.62, 95% CI: 0.01-42.36, p = 0.215). There was no statistically significant association between gender and mortality; however, male patients have increased odds of mortality (OR: 1.33, 95% CI: 0.16-16.87, p = 1.000) (Table 4).

**Table 4:** Associations between age, sex, and mortality SD: standard deviation, OR: odds ratio, CI: confidence interval

Variable	Category	Mortality (n = 7)	Alive (n = 23)	Statistical test	P-value	OR	95% CI
Age (years)	<60	1 (14.3%)	10 (43.5%)	Fisher’s exact	0.215	4.62	0.01-42.36
	≥60	6 (85.7%)	13 (56.5%)	-	-	-	-
	Mean ± SD	68.5 ± 6.9	56.4 ± 14.7	t-test	0.064	-	-
Sex	Male	5 (71.4%)	15 (65.2%)	Fisher’s exact	1.000	1.33	0.16-16.87
	Female	2 (28.6%)	8 (34.8%)	-	-	-	-

Associations between cardiovascular disease risk factors, comorbidities, and mortality

Although not statistically significant, there were increased odds of mortality associated with hypertension, alcohol intake, diabetes mellitus, and stroke (2.63, 2.50, 2.67, and 8.80, respectively) (Table 5).

**Table 5:** Associations between CVD risk factors, comorbidities, and mortality CVD: cardiovascular disease, OD: odds ratio, CI: confidence interval

CVD risk factors/comorbidities	Mortality (n = 7)	Alive (n = 23)	Statistical test	P-value	OR	95% CI
CVD risk factors						
Hypertension	6 (85.7%)	16 (69.6%)	Fisher’s exact	0.638	2.63	0.33-20.85
Alcohol intake	4 (57.1%)	8 (34.8%)	Fisher’s exact	0.392	2.50	0.42-14.85
Diabetes mellitus	2 (28.6%)	3 (13.0%)	Fisher’s exact	0.565	2.67	0.32-22.39
Dyslipidemia	0 (0%)	6 (26.1%)	Fisher’s exact	0.290	0.00	-
Smoking	0 (0%)	3 (13%)	Fisher’s exact	1.000	0.00	-
Heart failure	2 (28.6%)	9 (39.1%)	Fisher’s exact	1.000	0.62	0.08-4.71
Comorbidities						
Stroke	2 (28.6%)	1 (4.3%)	Fisher’s exact	0.128	8.80	0.71-108.86
CKD	0 (0%)	1 (4.3%)	Fisher’s exact	1.000	0.00	-

Association between ECG changes and mortality

The presence of reciprocal changes on the ST-segment on ECG was associated with increased mortality (p = 0.048). Although not statistically significant, ST-segment elevation (OR: 3.25), ST-segment depression (OR: 5.0), and T wave inversion (OR: 3.86) were associated with increased odds of mortality (Table 6).

**Table 6:** Association between ECG changes and mortality ECG: electrocardiogram, OR: odds ratio, CI: confidence interval, LV: left ventricle

ECG changes	Mortality (n = 7)	Alive (n = 23)	Statistical test	P-value	OR	95% CI
ST-segment elevation	5 (71.4)	10 (43.5)	Fisher’s exact	0.390	3.25	0.40-39.37
ST-segment depression	3 (42.9)	3 (13)	Fisher’s exact	0.120	5.00	0.5-275.5
Reciprocal changes	2 (28.6)	0 (0)	Fisher’s exact	0.048	25.0	1.06-588.5
T wave inversion	6 (85.7)	14 (60.9)	Fisher’s exact	0.372	3.86	0.35-196.8
LA enlargement	4 (57.1)	11 (47.8)	Fisher’s exact	1.000	1.45	0.19-12.12
LV enlargement	3 (42.9)	9 (39.1)	Fisher’s exact	1.000	1.17	0.14-8.78
Arrhythmia	1 (14.3)	6 (26.1)	Fisher’s exact	1.000	0.47	0.01-5.50

Association between echocardiogram findings and mortality

The association between echocardiogram findings and mortality in the study population is shown in Table 7. There were no significant associations between the echocardiogram findings and mortality in the study population.

**Table 7:** Association between echocardiogram findings and mortality EF: ejection fraction, LV: left ventricle, CI: confidence interval, OR: odds ratio *Adjusted chi-square Hyphens indicate non-applicable or not calculated values.

Echocardiogram findings	Mortality (n = 7)	Alive (n = 23)	Statistical test	P-value	OR	95% CI
Regional wall abnormality	4 (57.1%)	10 (43.5%)	Fisher’s exact	0.675	1.73	0.23-14.42
Ejection fraction	-	-	Fisher’s exact	1.000	0.00	0.00-6.15
Reduced	0 (0%)	2 (8.7%)	-	-	-	-
Preserved	7 (100%)	21 (91.3%)	-	-	-	-
LV geometry	-	-	χ² = 1.195*	0.754	0.82	0.14-4.79
Normal	0 (0%)	4 (17.4%)	-	-	-	-
Concentric remodeling	0 (0%)	6 (26.1%)	-	-	-	-
Concentric hypertrophy	3 (42.9%)	11 (47.8%)	-	-	-	-
Eccentric hypertrophy	1 (14.3%)	0 (0%)	-	-	-	-
TAPSE (<1.7 cm)	1 (14.3%)	2 (8.7%)	Fisher’s exact	1.000	1.75	0.03-38.62
Diastolic dysfunction	2 (33.3%)	13 (56.5%)	Fisher’s exact	0.390	0.31	0.03-2.47
Pericardial effusion	0 (0.0%)	1 (4.3%)	Fisher’s exact	1.000	0.00	0.00-7.51

Association between biochemical parameters and treatment outcome

There was no significant difference in the mean creatine (p = 0.357) and urea (p = 0.154) between survivors and those who died. The lipid parameters were similar between them, except LDL (p = 0.040), which was significantly elevated in individuals who died. Fasting blood glucose was similar between survivors and those who died (p = 0.623) (Table 8).

**Table 8:** Association between renal function, lipid profile, and fasting blood glucose and mortality in the study population Values are presented as mean ± SD. The independent samples t-test was used to compare continuous variables between mortality and survivor groups. P-values < 0.05 were considered statistically significant. Cr: creatine, TC: total cholesterol, LDL: low-density lipoprotein, HDL: high-density lipoprotein, TGA: triglyceride, FBG: fasting blood sugar, SD: standard deviation

Parameter (units)	Mortality (n = 7), mean ± SD	Alive (n = 23), mean ± SD	t-test (t)	P-value
Creatine (mg/dL)	1.0 ± 0.4	1.4 ± 1.1	0.877	0.357
Urea (mg/dL)	26.3 ± 10.0	48.9 ± 36.8	1.171	0.154
Total cholesterol (mg/dL)	237.7 ± 38.3	205.7 ± 35.5	1.592	0.126
LDL cholesterol (mg/dL)	178.1 ± 76.5	130.8 ± 36.3	2.164	0.040
HDL cholesterol (mg/dL)	39.7 ± 13.2	44.7 ± 12.0	-1.421	0.382
Triglycerides (mg/dL)	105.5 ± 57.0	128.2 ± 43.4	-0.878	0.296
Fasting blood glucose (mg/dL)	105.5 ± 12.8	109.0 ± 15.9	-0.118	0.623

Association between cardiac enzyme levels, acute coronary syndrome classification, and mortality

Elevated troponin level was associated with a statistically significant increase in mortality in the study population (p = 0.010). Mortality was higher in patients with STEMI compared to those with NSTEMI, with an odds ratio of 7.8, but this did not reach statistical significance (p = 0.086) (Table 9).

**Table 9:** Association between cardiac enzyme levels, ACS classification, and mortality OR: odds ratio, CI: confidence interval, CK-MB: creatine kinase-MB, ACS: acute coronary syndrome, STEMI: ST-segment elevation myocardial infarction, NSTEMI: non-ST-segment elevation myocardial infarction, UA: unstable angina

Parameter	Mortality (n = 7)	Alive (n = 23)	Statistical test	P-value	OR	95% CI
Cardiac enzymes						
Elevated troponin T	7 (100%)	12 (52.2%)	Fisher’s exact	0.010	12.0	1.00-143.2
Elevated CK-MB	6 (85.7%)	12 (52.2%)	Fisher’s exact	0.193	4.67	0.41-53.0
ACS classification						
STEMI	6 (85.7%)	10 (43.5%)	Fisher’s exact	0.086	7.80	0.71-385.7
NSTEMI	1 (14.3%)	13 (56.5%)	Fisher’s exact	0.086	0.14	0.01-1.42
UA	0 (0%)	9 (39.1%)	Fisher’s exact	0.086	0.00	0.00-0.74

## Discussion

Acute coronary syndrome (ACS) is associated with increased morbidity and all-cause mortality, varying by subtype and patient comorbidities. Although literature on ACS in Nigeria and sub-Saharan Africa is limited, the rising prevalence of cardiovascular risk factors suggests an increasing disease burden. This study aimed to bridge knowledge gaps relating to clinical parameters and treatment outcomes in our population.

The mean age of patients with ACS in this study was 59.0 ± 14.2 years, with most patients aged 60 years or older. This aligns with the findings of Sani et al., who reported similar age trends in a five-year review of ischemic heart disease at Aminu Kano Teaching Hospital [24]. Kolo et al. found a slightly lower mean age (55.6 ± 12.7 years) but within a comparable age range (40-82 years) [25]. Similarly, Affangla et al. in Senegal reported a mean age of 59 years among 52 patients with ACS [26]. Findings from the Kerala ACS Registry in India also revealed a similar mean age (60 ± 12 years) [27]. These consistent findings suggest a rising ACS burden among older adults, as seen globally.

The incidence of ACS was approximately twice as high in men compared to women, a trend observed across studies [24-26]. This gender difference is often attributed to a higher prevalence of risk factors such as hypertension and smoking among men.

The leading cardiovascular risk factors identified were hypertension, alcohol intake, dyslipidemia, and diabetes. Notably, alcohol intake ranked second in prevalence, higher than in other reports, suggesting changing lifestyle patterns in southern Nigeria. Excessive alcohol consumption is a recognized risk factor for ACS and adverse outcomes [28]. Smoking prevalence was relatively low in this cohort compared to prior reports from Sani et al. [24] and Kolo et al. [25].

The predominant clinical presentations included palpitations and chest discomfort in over 80% of patients, consistent with findings by Mihajlović et al. [29]. Atypical symptoms, including dyspepsia and heart failure, were also observed, aligning with the report of Sani et al., who documented left ventricular failure in 18.2% and dyspeptic symptoms in 9.1% of ACS cases [24].

Electrocardiographic patterns showed that 53% had ST-segment elevation, predominantly involving the inferior leads, followed by anteroseptal leads. Celik et al. [30] reported similar findings, whereas Sani et al. [24] and Falase et al. [18] found predominant anteroseptal and anterolateral involvement, respectively.

Echocardiographic findings commonly included regional wall motion abnormalities, abnormal left ventricular geometry, diastolic dysfunction, reduced ejection fraction, tricuspid regurgitation, and pericardial effusion. Mohanan et al. similarly reported that most patients with ACS had preserved systolic function [27].

STEMI was the most prevalent ACS type (53.3%), followed by unstable angina (30%) and NSTEMI (16.7%), consistent with Mihajlović et al. [29] and Mohanan et al. [27]. Mortality in this study was 23.3%, comparable to the 21% reported by Affangla et al. [26] and 22.7% by Sani et al. [24]. Lower rates have been observed in Kerala and Western cohorts [11,27,31].

Older patients (≥60 years) had higher observed mortality (OR: 4.62); however, this association did not reach statistical significance and should be interpreted cautiously given the small sample size and wide confidence interval, consistent with trends reported by Ulasi et al. [16] and Yusuf et al. [12]. Gender was not significantly associated with mortality, consistent with Udell et al. [32] and Oguoma et al. [17].

Mortality was more frequently observed among patients with comorbid risk factors, particularly hypertension, diabetes, heavy alcohol intake, and stroke, but these associations were not statistically significant. These trends, rather than definitive risk relationships, align with observations reported by Celik et al. [30] and Mohanan et al. [27]. Elevated cardiac troponin T levels were observed in all patients who died, suggesting a potential prognostic role. However, given the small cohort and limited follow-up, the strength of this association should be interpreted cautiously, in line with prior reports by Shah et al. [33] and Peacock et al. [34]. Higher LDL cholesterol among non-survivors paralleled earlier findings [35,36].

STEMI accounted for 86.7% of all deaths, followed by NSTEMI (14%), with no mortality among unstable angina cases. STEMI was associated with higher odds of mortality (OR: 7.8), although this did not reach statistical significance, and conclusions regarding risk should be considered exploratory. The median hospital stay was six days for non-survivors and eight days (IQR: 5-15) for survivors, consistent with Keller et al. [37] and Takada et al. [38].

The outcome of the study suggests that ACS in Nigeria shares similar epidemiological and clinical characteristics with other developing nations but occurs at relatively younger ages than in Western countries. The high short-term mortality observed likely reflects limited access to early reperfusion strategies (PCI and thrombolysis) and delayed presentation. The strong association between troponin T and mortality reinforces its prognostic importance, while the prominence of alcohol consumption as a risk factor highlights changing lifestyle dynamics.

This study has several limitations that should be considered when interpreting the findings. First, the relatively small sample size may have limited statistical power to detect modest associations, resulting in wider confidence intervals and reduced precision of effect estimates. Second, the study was conducted at a single tertiary care center, which may limit the generalizability of the results to other populations or healthcare settings. Third, the follow-up duration was limited to three months, restricting our ability to evaluate longer-term outcomes, such as recurrent cardiovascular events or late mortality. Collectively, these factors underscore the need for caution in extrapolating the findings beyond the study population and highlight the value of future multicenter studies with larger sample sizes and extended follow-up to validate and expand upon these results.

## Conclusions

This study demonstrated that acute coronary syndrome (ACS) predominantly affected older male patients, with hypertension, alcohol use, diabetes, and dyslipidemia as the most frequent risk factors. ST-segment elevation myocardial infarction (STEMI) was the most common presentation, followed by unstable angina and NSTEMI. Electrocardiographic findings frequently showed ST-segment elevation in inferior and anteroseptal leads, while echocardiography revealed diastolic dysfunction and regional wall motion abnormalities. Elevated troponin levels were observed in most patients and were strongly associated with mortality. The overall short-term mortality rate was 23.3%, highest among patients aged 60 years and above and those with STEMI. These findings emphasize that ACS in Nigeria remains a major cause of morbidity and mortality, necessitating improved preventive strategies, early diagnosis, and prompt reperfusion therapy to enhance patient outcomes.
